# Pneumococcal meningitis with normal cerebrospinal biochemistry and no pneumococci at microscopy, mimicking a stroke: a case report

**DOI:** 10.1186/s13256-017-1287-2

**Published:** 2017-06-07

**Authors:** Gideon Ertner, Jeppe Romme Christensen, Christian T. Brandt

**Affiliations:** 1 Department of Pulmonary and Infectious Diseases, Nordsjællands Hospital, University of Copenhagen, Hillerød, Denmark; 2 Department of Neurology, Rigshospitalet, University of Copenhagen, Copenhagen, Denmark

**Keywords:** Bacterial meningitis, Stroke, Neurological deficits, Invasive pneumococcal disease, Cerebrospinal fluid analysis and culture, Case report

## Abstract

**Background:**

Bacterial meningitis commonly presents with symptoms such as headache, impaired consciousness, neck stiffness, and fever. In most cases, cerebrospinal fluid analysis will yield white cell counts >100/mm^3^. Atypical presentations occur, especially in the very young or very elderly and the immunocompromised. We report an unusual case of pneumococcal meningitis in a healthy 78-year-old Danish woman who presented with clinical features mimicking a stroke with normal cerebrospinal fluid parameters and without microscopic evidence of bacteria.

**Case presentation:**

The patient was admitted after being found unconscious on her bed. Upon admittance, she was considered confused, with a temperature of 39.4 °C and slight neutrophilic leukocytosis, but no neck stiffness. A neurological examination revealed bilateral horizontal nystagmus, unstable eye movements, and suspected right-sided gaze paralysis. Cerebrospinal fluid analysis revealed normal parameters, and the microscopy result was negative for bacteria. The most likely diagnosis was considered to be stroke with concomitant infection. However, cerebrospinal fluid and blood cultures subsequently were rapidly positive for pneumococci. Neither immunodeficiency nor blood contamination was considered a likely cause of this discrepancy.

**Conclusions:**

This case emphasizes the need to consider a multidisciplinary approach and empirical meningitis treatment until diagnostic results from microbiological cultures are obtained.

## Background

Bacterial meningitis commonly presents with symptoms such as headache, impaired consciousness, neck stiffness, and fever. In a nationwide study in the Netherlands comprising 696 cases of bacterial meningitis, at least two of these symptoms were present in 95% of cases [1]. In the same study, cerebrospinal fluid (CSF) analysis yielded white cell counts >100/mm^3^ in 93% of cases. *Streptococcus pneumoniae* was the causative agent in 51% of cases with a positive CSF culture, and the causative agent was determined by Gram’s staining and microscopy of CSF in 80% of cases (with 97% specificity). Atypical presentations occur especially in the very young or very elderly and the immunocompromised [2]. We report an unusual case of pneumococcal meningitis in a healthy 78-year-old Danish woman who presented with clinical features mimicking a stroke with normal CSF parameters and without microscopic evidence of bacteria. However, CSF and blood culture results were rapidly positive for pneumococci.

## Case presentation

### Patient information

Our patient was a 78-year-old Danish woman with a history of hyperthyroidism for 15 years caused by a toxic multinodular goiter. The condition was uncomplicated and treated with thiamazole. She was also receiving statin therapy for hypercholesterolemia. She had had successful knee replacement surgery 7 years before the current admission. She lived alone, was fully mobile, and was not dependent on care.

In the 5 weeks prior to admission, she had had two episodes of syncope/presyncope. The first time she did not lose consciousness and attributed it to orthostatism. Her general practitioner ordered blood tests and referred her to an otorhinolaryngologist. The second episode happened 4 weeks prior to admission. She had been drinking two glasses of wine and subsequently felt dizzy and experienced loss of consciousness for up to 1 minute. She was immediately attended by family members, who did not observe convulsions or neurological deficits. She regained consciousness rapidly and was admitted to our hospital, where the results of a clinical examination, including a full neurological evaluation, routine blood tests, and electrocardiogram (ECG), were normal. A urine rapid test was positive for leukocytes and nitrite, and a subsequent culture revealed the presence of *Escherichia coli*. The patient was discharged the same day without treatment.

### Clinical presentation

The day prior to admission, family members could confirm that the patient was doing well and went to bed feeling well. A granddaughter was visiting the patient, and the next morning she found the patient lying unconscious on her bed as if she had collapsed and fallen backward onto the bed. The ambulance paramedics reported an initial Glasgow Coma Scale (GCS) score of 6. The patient was admitted to the emergency department, where she was examined by the doctor on call along with a consultant in infectious medicine. The patient was evaluated with a GCS score of 12. She was found febrile with a rectal temperature of 39.4 °C, but without neck stiffness. She had pinpoint pupils and brief twitching of the left-side extremities but no convulsions. The patient was considered confused, unable to recall her date of birth, but she could otherwise respond with a whispering voice. She denied complaints of headache or other pain.

### Diagnostic assessment

The result of a 12-lead ECG was normal, as was continuous ECG monitoring. The patient had no cardiac murmurs or clinical findings suggestive of pneumonia. Her ear examination was without signs of otitis.

Blood tests revealed marginal leukocytosis with 9.35 × 10^9^/L neutrophils. All other biochemistry results were normal, including a C-reactive protein level of 4 mmol/L. Arterial blood gas analysis revealed slight hyperventilation with a partial pressure of carbon dioxide of 4.0 kPa. The patient’s lactate level was 0.7 mmol/L.

On the basis of primary suspicion of stroke, the patient was seen by the neurologist on call, who found a bilateral horizontal nystagmus, unstable eye movements, and suspected right-sided gaze paralysis. On suspicion of a stroke affecting the pons, a computed tomographic scan including angiography was performed 2.5 hours after admission and was described as without signs of stroke or occlusions. Lumbar puncture was performed 4.5 hours after admission. The patient’s CSF pressure was 8 cm H_2_O, and the result of CSF analysis was normal (leukocyte cell count 4 × 10^6^/L, glucose 4.4 mmol/L, protein 0.23 g/L, no erythrocytes). CSF samples were sent immediately for microscopic analysis, the result of which was negative for bacteria. Meningitis was considered highly unlikely, and the patient was transferred to the neurology department, where 5.5 hours after admittance she was started on empirical sepsis treatment with cefuroxime 1500 mg three times daily. After 7 hours, treatment with clopidogrel and acetylsalicylic acid was initiated.

The next morning, 22 hours after the patient had been found unconscious, three of three CSF cultures yielded growth of pneumococci. A few hours later, blood culture results also became positive for pneumococci. The patient was stable and fully conscious with only minimal confusion.

### Treatment

Upon the discovery of pneumococci in the cultures, the patient was immediately started on ceftriaxone 4 g once daily, 3 million U of benzylpenicillin six times daily, and dexamethasone 10 mg four times daily. The pneumococci were penicillin-susceptible, and the patient was treated for 14 days and discharged after 16 days. The course of disease was uncomplicated, except for influenza confirmed by polymerase chain reaction on day 10 of admittance. The patient’s influenza was treated with oseltamivir phosphate (Tamiflu; Genentech, South San Francisco, CA, USA) 75 mg twice daily.

### Outpatient evaluation

The patient was seen in the outpatient clinic 8 days after discharge, where her daughter complained of worsening of her mother’s hearing deficit, impaired balance, and fatigue. The patient was referred to an otorhinolaryngologist. Audiometry revealed a bilateral moderate perceptive hearing loss. The patient’s impaired balance was described as a long-standing problem with only minor aggravation after the admission.

**Fig. 1:**
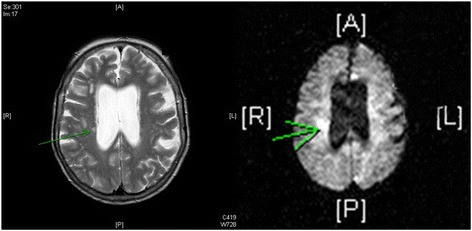
Magnetic resonance imaging scan showing the suspected infarction in the right corona radiata, *green* arrows (*left*: T2-weighted image, *right*: diffusion-weighted image)

The patient’s neurological examination result was normal, apart from marginally unstable walking and hyperreflexia of the left upper extremity. A brain magnetic resonance imaging scan showed brain atrophy with ventricular enlargement and, on the basis of diffusion-weighted images, a small brain infarction in the right corona radiata was suspected (Fig. 1). The patient was recommended prophylactic antihypertensive and antithrombotic therapy and pneumococcal vaccination, and she was discharged to the care of her general practitioner 3 months after hospital discharge. Shortly after discharge, the patient resumed all daily activities. She had no memory of the day of admittance but remembered she had felt cold and shivering before going to bed the night before admission. She also remembered getting up in the morning to find her bathrobe because she was feeling cold when she remembers suddenly falling backward.

## Discussion

The present case reminds us of two rare presentations of bacterial meningitis in the elderly and immunocompromised, namely the strokelike presentation and meningitis in the absence of CSF inflammation. It is well known that a few patients with bacterial meningitis present with normal CSF parameters [3]. In these cases, however, bacteria are normally found by microscopy, suggesting severe disease with compromised immune capacity [4]. Although our patient was quite healthy, it is known that a range of age-related changes occur in the immune systems of the elderly that may cause varying degrees of immune deficiency [5].

The combination of a previous episode of unexplained dizziness and collapse together with a new episode suspicious of sudden collapse and discrete neurological deficits suggested stroke as a potential cause of disease. The only initial finding suggestive of ensuing infection was a temperature above 39 °C and slight neutrophilic leukocytosis. Despite the patient’s recollecting that she had actually had symptoms suggestive of infection more than 10 hours prior to admission, her CSF and blood biochemistry results were normal, apart from her blood neutrophil counts.

The significant clinical improvement the patient experienced following treatment with cefuroxime suggests that this drug may have had sufficient penetration into the CSF to exceed the minimal inhibitory concentration of pneumococci or that the elimination of the concurrent bacteremia was of similar benefit because the local brain compartment and systemic compartment are well described to be closely related [4, 6].

The episodes of syncope/presyncope 4 and 5 weeks before admission were most probably caused by a minor urinary tract infection. For unclear reasons, this was not treated, but we find it unlikely to have influenced the cause of disease, because the patient experienced no symptoms during the 4 intervening weeks.

## Conclusions

This case emphasizes the need for a multidisciplinary approach to complex cases and also that empirical antibiotic therapy and even empirical meningitis treatment may be appropriate until diagnostic results of microbiological cultures are obtained.

## Abbreviations

CSF: Cerebrospinal fluid
ECG: Electrocardiogram
GCS: Glasgow Coma Scale
